# Exploring the dynamics of FGF23 in patients with Hereditary Hemochromatosis type I following iron depletive treatment: a pilot study

**DOI:** 10.1007/s40618-026-02893-5

**Published:** 2026-05-26

**Authors:** Luciano Colangelo, Sergio Terracina, Chiara Sonato, Viviana De Martino, Giancarlo Ferrazza, Enrico Panzini, Daniele Diacinti, Luciano Nieddu, Francesca Arienzo, Marco Occhiuto, Cristiana Cipriani, Stefania Trasarti, Antonio Angeloni, Jessica Pepe, Alessandro Corsi, Salvatore Minisola

**Affiliations:** 1 Department of Medical and Cardiovascular Sciences, Sapienza University, Rome, Italy; 2 Department of Experimental Medicine, Sapienza University, Rome, Italy; 3 Department of Immunohaematology and Transfusion Medicine, Sapienza University, Rome, Italy; 4 Department of Radiological Sciences, Oncology and Pathology, Sapienza University, Rome, Italy; 5 Department of Humanistic and International Social Sciences, UNINT University, Rome, Italy; 6 Pathology Unit, Bambino Gesù Children’s Hospital, IRCCS, Rome, Italy; 7 Department of Translational and Precision Medicine, Sapienza University, Rome, Italy; 8 Department of Molecular Medicine, Sapienza University, Rome, Italy

**Keywords:** Hereditary Hemochromatosis, *HFE*, Iron status, iFGF23, cFGF23, Phosphate, Homeostasis, Osteopenia

## Abstract

**Purpose:**

Anemia, inflammation and iron deficiency are linked to Fibroblast growth factor 23 (FGF23). Aim of this study was to explore the dynamics of FGF23 in Hereditary Hemochromatosis type-I (HH1).

**Methods:**

Twenty-six consecutive patients with genetically confirmed and uncomplicated HH1 and nineteen healthy age-matched voluntary blood donors (CTR) were enrolled for the study. Intact (iFGF23) and C-terminal (cFGF23) FGF23 and iron status markers were evaluated at baseline (T0) and seven days (T7) after phlebotomy/voluntary blood donation (VBD). Bone mineral density (BMD) and vertebral fracture assessment (VFA) were also evaluated at T0.

**Results:**

Cross-sectional and longitudinal analyses failed to reveal significant differences in iFGF23 in both HH1 (T0: 54.27 ± 14.42 pg/mL; T7: 54.70 ± 15.48 pg/mL) and CTR (T0: 52.77 ± 17.63 pg/mL; T7: 53.27 ± 15.88 pg/mL) groups. Absence of significant difference was also observed for cFGF23 at baseline between the two groups (HH1, T0: 0.98 ± 0.39 pmol/L; CTR, T0: 1.18 ± 0.91 pmol/L) but not at T7 when the values (HH1, T7: 01.20 ± 0.89; CTR, T7: 1.84 ± 1.11 pmol/L) were significantly increased in CTR compared to T0 (p = 0.0003) and to HH1 (p = 0.0240). After phlebotomy/VBD, in both HH1 and CTR groups, serum levels of phosphate were unchanged from baseline while serum iron, ferritin and transferrin saturation and erythrocyte-related parameters (red blood cells, hemoglobin and hematocrit) were significantly reduced (all p  <  0.0001). Serum iron, ferritin, and transferrin saturation were significantly higher in HH1 than in CTR (p = 0.0002 for serum iron and p  <  0.0001 for both ferritin and transferrin saturation) at T7. In the CTR group, these parameters showed a trend toward iron deficiency whereas in HH1 they remained near the upper limit of the normal range, suggesting a persistent mild iron overload. Correlation and regression analyses did not show significant associations between circulating FGF23 (both iFGF23 and cFGF23) and iron status markers. BMD and VFA were not significantly different between HH1 and CTR. Furthermore, BMD and Trabecular Bone Score values were not associated with circulating FGF23 levels.

**Conclusion:**

This pilot study indicates that the serum levels of iFGF23 and cFGF23 and skeletal health evaluated through BMD and VFA do not differ between patients with uncomplicated HH1 and healthy subjects at baseline. The absence of changes in the serum levels of iFGF23 and cFGF23 in HH1 patients with uncomplicated HH1 after phlebotomy may reflect the persistence of iron overload which could counteract the physiological hypoxia-driven stimulation of FGF23 production and cleavage observed in healthy subjects after VBD.

## Introduction

Fibroblast growth factor 23 (FGF23) is a key regulator of phosphate homeostasis [1, 2]. It is mainly produced in bone, decreases renal NPT2a (sodium phosphate co-transporter 2a), NPT2c (sodium phosphate co-transporter 2c) and vitamin D 1α-hydroxylase expression while stimulates 24-hydroxylase expression, resulting in decreased renal phosphate reabsorption and decreased serum phosphate and 1,25-dihydroxyvitamin D (1,25D) concentrations [1, 2]. FGF23 production is regulated at transcriptionally and post-translational stages and before secretion it may undergo to proteolytic cleavage that abolishes the biological activity of full length intact FGF23 (iFGF23) and generates C-terminal and N-terminal fragments [1–3]. Consequently, the serum levels of bioactive FGF23 are determined by the balance between factors affecting transcription and post-translational cleavage. The availability of two complementary assays to detect iFGF23 and C-terminal FGF23 (cFGF23) provides valuable tools to investigate the FGF23 dynamics non-invasively [1, 4, 5].

Recent findings demonstrated that, in addition to phosphate, calcium, vitamin D, and parathyroid hormone [1, 2], FGF23 production and cleavage is regulated by other factors including anemia, inflammation and iron deficiency [1, 6–8]. Iron deficiency was first discovered to regulate FGF23 when it was recognized that patients with Autosomal Dominant Hypophosphatemic Rickets (ADHR) developed flares of hypophosphatemia that coincided with states of iron deficiency [9]. In addition, mice fed a low iron diet have increased FGF23 levels [10, 11] and inverse relationships between markers of iron status and FGF23 levels have been observed in some human cohorts [12, 13]. Iron deficiency, as also inflammation and erythropoietin (EPO), stimulates *FGF23* transcription and increases FGF23 cleavage with the result that the serum levels of cFGF23 increase and those of iFGF23 and phosphate are maintained in the normal range [1, 8]. Iron chelators have been shown to stimulate FGF23 production in bone cell cultures [8, 14] while increased FGF23 proteolytic cleavage during iron deficiency has been reported to be, at least partially, mediated by Hypoxia-Inducible Factor (HIF)1α-dependent upregulation of furin, which is known to cleave iFGF23 [15, 16].

While the studies regarding the relationship between iron deficiency and FGF23 have been grown in the last years, those aimed to evaluate the relationship between iron overload conditions and FGF23 are limited and mainly focused on Beta-Thalassemia (βT). In a murine model of intermedia βT, which spontaneously develop iron overload on a standard iron diet, serum levels of iFGF23 and cFGF23 were both significantly increased compared to WT mice [17]. In humans, data on serum levels of FGF23 in βT showed conflicting results across studies likely reflecting the heterogeneity of the evaluated population (e.g., children and adolescents vs. adults, disease severity, chelation therapy or not, transfusion dependency or not) as also the variations in the assay methodologies [18].

Hereditary Hemochromatosis type 1 (HH1, OMIM #235200) is an inherited recessive disorder characterized by progressive iron accumulation in different tissues and organs potentially leading to chronic damage and dysfunction [19]. The first line treatment in HH1 patients is phlebotomy [19, 20]. Although HH1 patients may develop bone alterations, especially osteopenia and osteoporosis, the relationship between HH1 and bone health remains relatively underexplored [21–24]. Notably, no data do exist regarding the serum levels of FGF23 in HH1. Moreover, most of the studies have been conducted in patients with advanced disease and complications (e.g., cirrhosis, hypogonadism, diabetes and cardiac dysfunction) that can have an impact on bone health per se [25]. Thus, distinguishing in the skeleton the direct impact of iron overload from the secondary effects related to these complications represents a significant challenge.

The aim of this pilot study was to investigate whether the increased iron absorption and the consequent iron overload in HH1 patients without organ dysfunction based on clinical, biochemical and instrumental investigations and on induction therapy with phlebotomy has an impact on the serum levels of iFGF23 and cFGF23 and on the skeleton. By performing this study, we aim to start to explore the effects of HH1-related iron overload on the dynamics of FGF23 and on the skeletal health of uncomplicated HH1.

## Methods

### Study design

The study was conducted in accordance with the Declaration of Helsinki and approved by the Ethics Committee of “Sapienza” University of Rome (protocol number 0374/2022). Written informed consent was obtained from all the participants.

We enrolled from 1st October 2021 to 30th November 2022, twenty-six consecutive patients with genetically confirmed HH1 who had no evidence of clinical, biochemical and instrumental organ dysfunction including cirrhosis, hypogonadism, diabetes and cardiomyopathy and were on the induction phase of treatment through phlebotomy. HH1 patients with long-standing disease and related complications due to iron overload, and/or those receiving iron chelation therapy, were excluded.

Nineteen age-matched healthy volunteer blood donors were also recruited as control group (CTR) and evaluated at the same time points of HH1 patients. Criteria of exclusion of both HH1 patients and healthy donors included also the identification of reduced estimated glomerular filtration rate (eGFR, < 60 mL/min/1.73m^2^), hyperglycemia, elevated serum levels of transaminases and clotting factors abnormalities at the time of phlebotomy/voluntary blood donation (VBD). Both HH1 patients and healthy donors were evaluated at baseline (i.e., the day of therapeutic phlebotomy for HH1 patients and voluntary blood donation for healthy subjects) and after one week (T7). The seven-day interval was chosen with the specific aim to capture early changes in the dynamics of FGF23 in response to blood depletion and to investigate on a short-term regulatory response, while minimizing the influence of long-term compensatory mechanisms [9, 12, 26, 27].

### Blood sampling

Blood samples were collected between 8:00 and 9:00 from each HH1 patient and healthy donor at T0 and T7 after a fasting period of at least 10 h. Samples were stored at -70 °C and finally analyzed in one batch at the end of the study. Conventional biochemical parameters were assayed as previously described [28]. Additionally, iron status markers, namely serum levels of iron, ferritin and transferrin and transferrin saturation (TSAT), and iFGF23 and cFGF23 were also measured. For each HH1 patient and healthy donor, TSAT was calculated according https://www.emocromatosi.it/test.asp. Serum ionized calcium (Ca^++^) was measured using an ion-specific electrode (Nova 8; Nova Biochemical, Waltham, MA). Kidney function was estimated using the abbreviated Modification of Diet in Renal Disease (MDRD) formula to calculate the eGFR. iFGF23 and cFGF23 concentrations were measured by radioimmunoassay (RIA) (Diasorin Inc., Stillwater, MN, USA) and an ELISA kit (BIOMEDICA, BI-20702, Vienna, Austria) respectively. For both assays, intra- and inter-assay coefficients of variation were 9% and 7.5% respectively.

### History of fractures, Bone Mineral Density (BMD), Trabecular Bone Score (TBS) and Vertebral Fracture Assessment with Vertebral Morphometry (VFA-MXA)

At baseline (T0), past fracture history was registered and BMD, TBS and VFA-MXA evaluated for each HH1 patient and healthy donor. BMD was measured using dual-energy X-ray absorptiometry (DXA, Lunar iDXA, GE Healthcare) at the lumbar spine (L1-L4), femoral neck (FN), total hip (TH) and in the distal third of the non-dominant radius. VFA-MXA was measured as previous described [29] and the severity of vertebral fractures was established according to Genant’s criteria [30]. All imaging examinations were performed by an experienced radiologist (D.D.) blinded to the study group assignment.

### Statistical analysis

Continuous variables were summarized as mean ± SD or median (interquartile range, IQR), according to distribution. For categorical variables, comparisons between groups were performed using the chi-square test. Normality of data was checked with Shapiro-Wilk and confirmed by Q-Q plot inspection. For independent groups, *t*-test was used when data for both groups were approximately normally distributed or the Mann-Whitney U test when normality assumption failed. For paired data (T0 vs. T7), the paired t-test was used if the distribution of within-subject differences was normal; otherwise, the Wilcoxon matched-pairs test was used. As iron status and erythropoiesis are involved in the regulation of FGF23 production and cleavage [1, 11, 31] and bone health may be impaired in HH1 patients [21–25], we assessed in both groups the correlations between iron status markers and serum levels of iFGF23 and cFGF23 at both T0 and T7 as well as the relationships between BMD and TBS and iron status markers and serum levels of iFGF23 and cFGF23 at T0. Pearson or Spearman correlation analysis was applied, as appropriate. To investigate pairwise relationships between variables, multiple regression models with appropriate interaction terms were applied to assess whether the relationships between HH1 patients and healthy subjects differed significantly. All tests were two sided with α = 0.05. P values < 0.05 were considered statistically significant (reported as *p* < 0.0001 when appropriate). Power calculation was carried out using Cohen’s d effect sizes (α = 0.05, two-tailed) with the final sample sizes (*n* = 26 for HH1 patients and *n* = 19 for healthy subjects). Power was around 0.8 or higher for large effect sizes (d = 0.8) while for medium effect size (d = 0.5) the power dropped to 0.4–0.6. Statistical analyses were performed using GraphPad Prism Software (version 10.4.1, GraphPad. Software Inc., San Diego, CA, USA).

## Results

This study includes 26 HH1 patients (M: F = 24/2, median age 42.00 years, IQR: 33.00–52.00 years) and 19 blood volunteer healthy donors (M: F = 17/2, median age 49.00 years, IQR: 27.00–52.00 years). All HH1 patients tested positive for common variants in the *HFE* gene [19], specifically C282Y homozygosity (57.7%), C282Y/H63D compound heterozygosity (27.3%) and homozygosity for H63D (15%) and performed at least one phlebotomy before the enrollment (mean *±* SD 3.60 *±* 3.53, median 2, range 1–15). The unique or last phlebotomy was performed 11.22 *±* 15.24 months (median 8 months and range 2–72 months) before the enrollment. Table 1 shows the demographic and anthropometric parameters and laboratory values in both groups.

**Table 1:** Demographic and anthropometric parameters and biochemical values at baseline (T0) and after phlebotomy/blood donation (T7) in HH1 and CTR groups

	HH1		CTR		Significance
Age (yrs)	43.81 ± 10.60		42.42 ± 13.16		ns
Population (n)	26		19		-
Gender (M: F)	24:2		17:2		ns
BMI (Kg/m^2^)	26.40 ± 3.47		24.83 ± 3.02		ns
Waist circumference (cm)	96.96 ± 9.35		93.11 ± 9.18		ns
History of fractures	5/26 (19%)		1/19 (5%)		ns
	**T0**	**T7**	**T0**	**T7**	
Days after phlebotomy/blood donation	6.96 ± 0.92		6.58 ± 0.77		ns
Serum Cr (mg/dL)	0.94 ± 0.14	0.93 ± 0.16	0.98 ± 0.13	0.99 ± 0.13	ns
Clearance Cr MDRD (n.v. >60 mL/min/1.73 m²)	93.91 ± 15.22	95.37 ± 17.18	89.02 ± 11.49	87.24 ± 14.08	ns
Serum iron (n.v. 64.80–174.90 mcg/dL)	155.80 ± 53.44	114.40 ± 43.59	100.10 ± 20.41	71.02 ± 25.74	* # ° *§*
Serum transferrin (n.v. 2.15–3.65 g/L)	2.58 ± 0.48	2.53 ± 0.44	2.89 ± 0.45	2.92 ± 0.34	# *§*
Serum ferritin (n.v. 30–300 mcg/L)	387.80 ± 313.70	296.42 ± 235.62	77.21 ± 67.20	52.11 ± 49.40	* # ° *§*
TSAT (n.v. 20–45%)	43.08 ± 16.33	32.23 ± 13.59	24.37 ± 5.10	17.00 ± 7.13	* # ° *§*
Serum calcium (n.v. 8.4–10.2 mg/dL)	9.91 ± 0.33	9.64 ± 0.38	9.89 ± 0.41	9.59 ± 0.36	*°
Ca^++^ (n.v. 1.17–1.33 mmol/L)	1.26 ± 0.04	1.27 ± 0.04	1.21 ± 0.05	1.23 ± 0.05	# *§*
Serum phosphate (n.v. 2.5–4.5 mg/dL)	3.27 ± 0.43	3.23 ± 0.50	3.27 ± 0.56	3.49 ± 0.60	ns
25(OH)D (n.v. 20–50 ng/mL)	25.27 ± 7.44	NA	33.05 ± 13.14	NA	#
PTH (n.v. 15–65 pg/mL)	44.32 ± 11.89	NA	42.52 ± 15.57	NA	ns
ALP (n.v. 40–129 IU/L)	70.46 ± 18.96	NA	68.84 ± 14.46	NA	ns
Hb (gr/dL)	15.60 ± 1.01	14.49 ± 0.94	15.21 ± 1.23	14 0.04 ± 1.48	* °
RBCs (x10^6^/mm^3^)	5.13 ± 0.41	4.66 ± 0.37	5.19 ± 0.39	4.77 ± 0.52	* °
HTC (%)	48.04 ± 3.14	43.13 ± 3.19	47.63 ± 3.73	43.57 ± 4.26	* °
iFGF23 (n.v. < 95 pg/mL)^	54.27 ± 14.42	54.70 ± 15.48	52.77 ± 17.63	53.27 ± 15.88	ns
cFGF23 [n.v., median (interquartile ranges), 0.88 (0.57–1.25) pmol/L]^^	0.98 ± 0.39	1.20 ± 0.89	1.18 ± 0.91	1.84 ± 1.11	° *§*

Data are expressed as mean *±* SD. HH1: Hemochromatosis type 1. CTR: healthy subjects, control group. Cr: creatinine; Fe: serum iron; TSAT: transferrin saturation; Ca^++^: serum ionized calcium; 25(OH)D: 25(OH) vitamin D; PTH: parathyroid hormone; ALP: alkaline phosphatase; Hb: haemoglobin; RBCs: red blood cells; HTC: haematocrit; NA: not available. ^according to references [44] and [45]; ^^ according to reference [46]. ^*^HH1-T0 vs. HH1-T7; ^#^HH1-T0 vs. CTR-T0; ^°^CTR-T0 vs. CTR-T7; ^§^HH1-T7 vs. CTR-T7. Specific p values are reported within the text

## Cross-sectional data

**Fig. 1:**
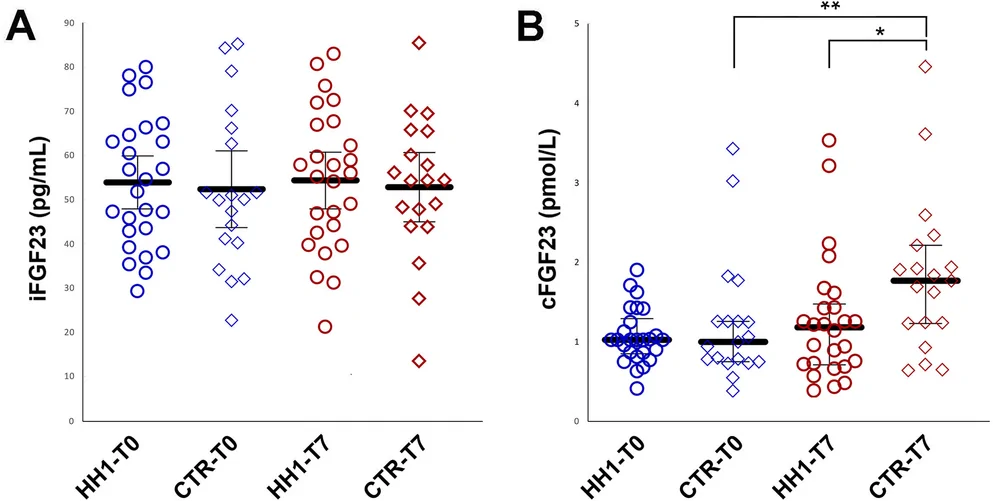
Scatter plots showing the serum levels of iFGF23 (**A**) and cFGF23 (**B**) in HH1 patients (HH1) and healthy subjects (CTR) at baseline (T0, blue) and seven days after phlebotomy/VBD (T7, red). Each circle identifies a HH1 patient and each diamond a healthy subject. Horizontal bars represent the mean with 95% confidence intervals. Compared to the HH1 group, the CTR group exhibited a significant increase in cFGF23 (* is for *p* = 0.0240) at T7. Longitudinal analysis within the CTR group further revealed a significant increase in cFGF23 (** is for *p* = 0.0019) at T7 compared to T0

At baseline (T0), no significant differences were observed for age, gender, BMI (HH1: median 25.40 Kg/m^2^, IQR: 24.18–27.83 Kg/m^2^; CTR: median 24.00 Kg/m^2^, IQR: 22.30–27.40 Kg/m^2^), waist circumference (HH1: median 95.00 cm, IQR: 88.75–102.30 cm; CTR: median 91.00 cm, IQR: 84.00–103.00 cm) and history of fractures between the two groups. The most common site of fracture referred by both HH1 patients and healthy donors was the distal radius. Albeit non significantly, they were more commonly registered in HH1 patients compared to healthy donors (*n* = 5/26 patients in HH1 and *n* = 2/19 in CTR). As expected, the HH1 group had significant higher mean values of biochemical parameters reflecting iron status in comparison with the control group (*p* < 0.0001 for serum iron, ferritin and TSAT). No statistically significant differences were observed in the serum levels of iFGF23 and cFGF23 (Table 1 and Fig. 1).

BMD (including the corresponding Z scores) and TBS values are shown in the supplementary material (Table 2**)**. Both BMD and TBS were not significantly different between HH1 patients and healthy donors. Additionally, vertebral fractures, were more frequently, albeit not significantly, detected in HH1 patients compared to healthy donors (*n* = 5/26 patients in HH1 and *n* = 1/19 in CTR). All vertebral fractures were classified as mild (20–25%) according to Genant’s criteria [30].

**Table 2:** BMD and TBS at baseline (T0) in HH1 and CTR groups

BMD (g/cm^2^)		HH1	CTR	Significance
	Lumbar spine	1.201 ± 0.158	1.249 ± 0.127	ns
	Femur neck	0.974 ± 0.110	1.045 ± 0.153	ns
	Total hip	1.040 ± 0.114	1.090 ± 0.143	ns
	Distal third of the non-dominant radius	0.958 ± 0.090	0.947 ± 0.096	ns
**Z score**				
	Lumbar spine	-0.376 ± 1.202	0.273 ± 1.007	ns
	Femural neck	-0.403 ± 0.881	0.200 ± 0.923	ns
	Total Hip	-0.196 ± 0.928	0.315 ± 0.873	ns
	Distal third of the non-dominant radius	-0.157 ± 0.661	-0.131 ± 0.924	ns
**TBS**		1462 ± 112	1493 ± 86	ns

BMD: Bone Mineral Density. TBS: Trabecular Bone Score

HH1 patients and healthy subjects were re-evaluated about one week (median and range 7 and 5–10 days in HH1 and 7 and 5–8 days in healthy subjects) after phlebotomy/VBD (p not significant). The HH1 group had significantly greater mean values of iron status markers in comparison with the CTR group (*p* = 0.0002 for serum iron and *p* < 0.0001 for both ferritin and TSAT). No statistically significant difference was observed in the serum levels of iFGF23 in both groups (Table 1 and Fig. 1). In contrast, in the CTR group, the serum levels of cFGF23 were significantly greater (*p* = 0.0240) compared to the HH1 group (Table 1 and Fig. 1).

### Longitudinal data

**Fig. 2:**
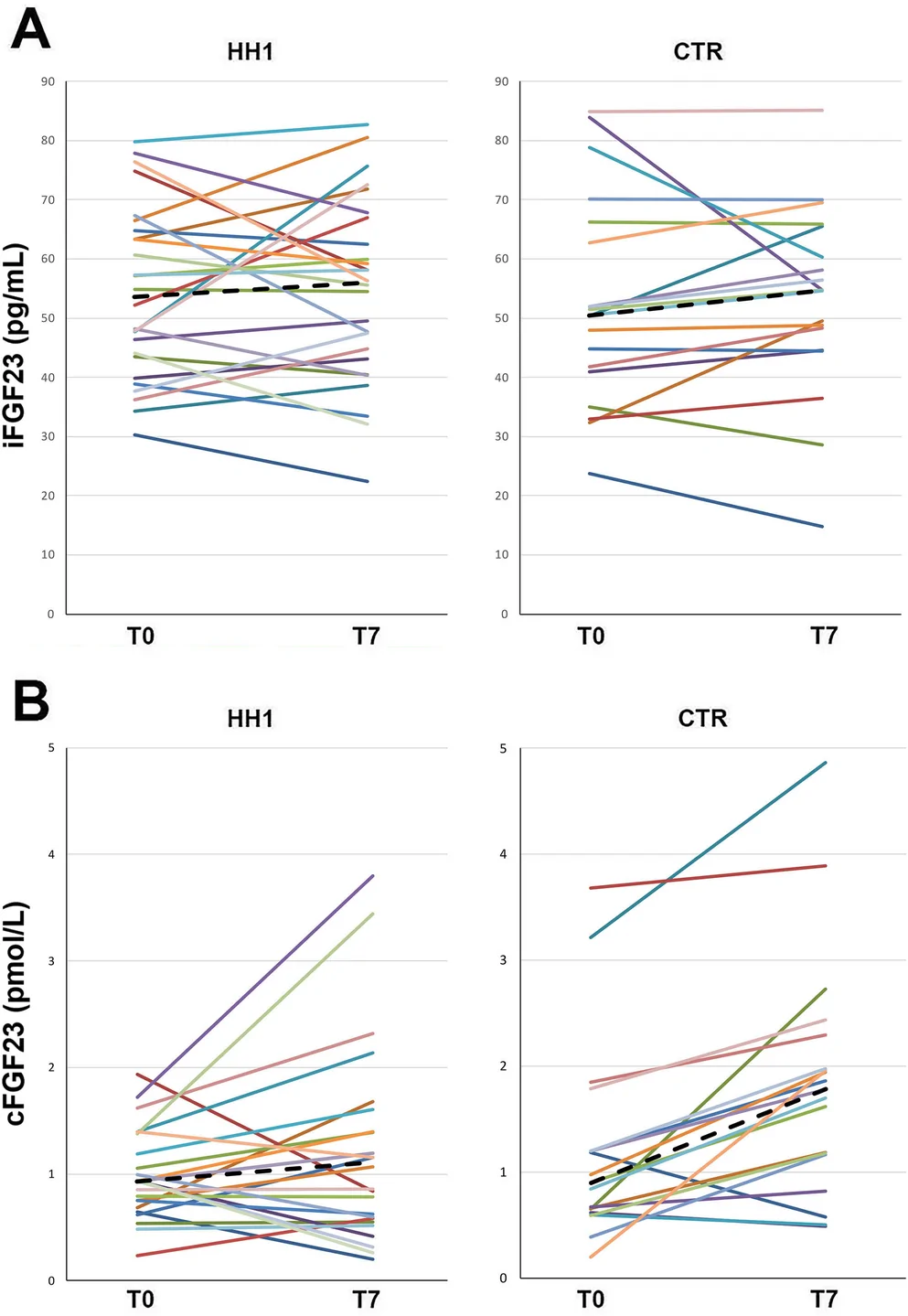
Measurements of iFGF23 (**A**) and cFGF23 (**B**) for each HH1 patient (HH1) and for each healthy donor (CTR) at baseline (T0) and seven days after phlebotomy/VBD (T7). Each HH1 patient and healthy donor is identified by a different color. The dashed black line in each graph connects the median values at T0 and T7

In both HH1 and CTR groups (Table 1), phlebotomy/VBD was associated with a significant reduction of Fe (HH1, T0 vs. T7 *p* = 0.0002; CTR, T0 vs. T7 *p* = 0.0001), Ferritin (HH1 and CTR, T0 vs. T7 *p* < 0.0001) and TSAT (HH1, T0 vs. T7 *p* = 0.0002; CTR, T0 vs. T7 *p* < 0.0001). HH1 and CTR groups shared also a significant reduction in red blood cells (RBCs), Hemoglobin (Hb) and Hematocrit (HCT) (for all parameters T0 vs. T7 *p* < 0.0001 in both groups). In both groups, significant change was also observed for serum calcium (HH1, T0 vs. T7 *p* = 0.0013; CTR, T0 vs. T7 *p* = 0.0028). Measurements of the serum levels of iFGF23 and cFGF23 for each HH1 patient and healthy donor at T0 and T7 are shown in Fig. 2. No significant difference was observed in the serum levels of iFGF23 between T0 and T7 in both HH1 patients and healthy subjects (Table 1 and Fig. 1). In contrast, while in healthy subjects the serum levels of cFGF23 were significantly increased after VBD (T0 vs. T7 *p* = 0.0003), they were unchanged in HH1 after phlebotomy (Table 1 and Fig. 1).

## Correlations and regression analyses

Correlation analyses did not reveal significant associations between either iFGF23 or cFGF23 and serum iron, ferritin, transferrin and TSAT in HH1 patients and healthy subjects at both T0 and T7. In regression models including group and interaction terms, none of the iron status markers were significantly associated with iFGF23, and all models showed very low explanatory power (R² < 0.07) indicating that iron markers do not contribute to the variability of circulating iFGF23. For cFGF23, no consistent associations were observed, except for a positive relationship with transferrin in the global regression model (β = +0.41, *p* = 0.048), which was not confirmed by subgroup analyses.

**Fig. 3:**
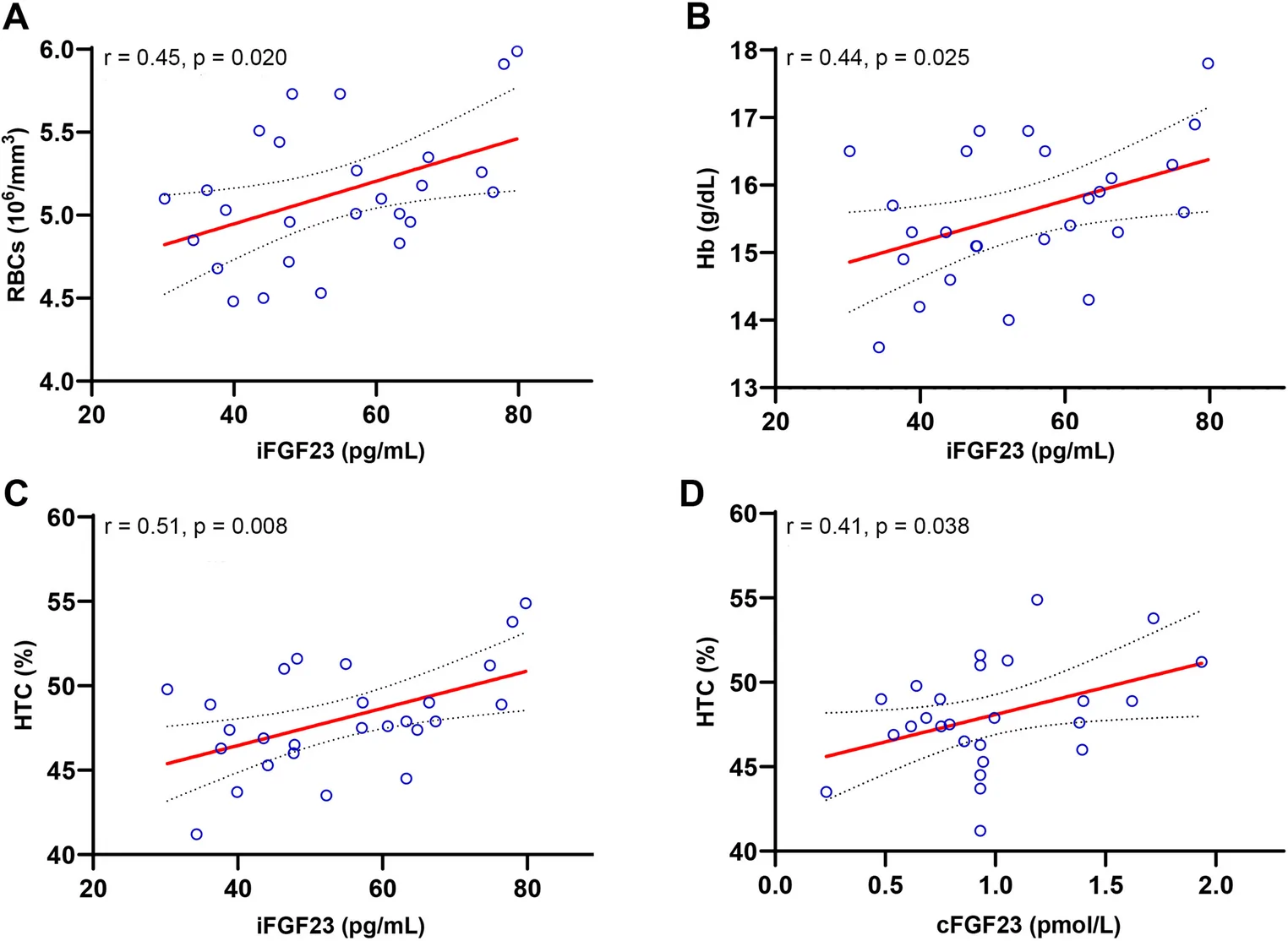
Scatter plots showing the significant correlations of iFGF23 with RBCs (**A**), Hb (**B**) and HCT (**C**), and of cFGF23 with HCT (**D**) in HH1 patients at baseline (T0). Each patient is represented by a blue circle. The solid red line indicates the linear regression fit and the dotted lines represent the 95% confidence interval

At baseline (T0), significant positive correlations were observed in HH1 patients between iFGF23 and RBCs (*r* = 0.45, *p* = 0.020), Hb (*r* = 0.44, *p* = 0.025) and HTC (*r* = 0.51, *p* = 0.008), and between cFGF23 and HTC (*r* = 0.41, *p* = 0.038) (Fig. 3). These associations were no longer present at T7 and were absent in healthy subjects. In the HH1 group, correlation analysis also revealed significant positive associations between the magnitude of the changes of erythrocyte-related parameters (ΔRBCs, ΔHb and ΔHCT) and that of iFGF23 levels (ΔiFGF23) between T0 and T7 (ΔRBCs: ρ = 0.44, *p* = 0.024; ΔHb: ρ = 0.42, *p* = 0.032; ΔHCT: ρ = 0.61, *p* = 0.001) indicating that smaller reductions in erythrocyte-related parameters were associated with greater increases in iFGF23. No significant correlations were observed between ΔcFGF23 and ΔRBCs, ΔHb, or ΔHCT in the HH1 group. Similarly, in the CTR group, neither ΔiFGF23 nor ΔcFGF23 showed significant correlations with ΔRBCs, ΔHb, or ΔHCT. Furthermore, when regression models including group and interaction terms were applied, no consistent associations were confirmed for either iFGF23 or cFGF23.

In HH1 patients and in healthy subjects, BMD values at the each of the evaluated sites (lumbar, neck, total hip and distal third of the non-dominant radius) and TBS showed no significant correlations with either iFGF23 or cFGF23 as also with iron status markers and haematological parameters. Regression analyses confirmed these findings, showing consistently low explanatory power (R² < 0.10) and non-significant coefficients. Overall, BMD and TBS do not appear to be influenced by circulating iFGF23, cFGF23, iron status markers and haematological parameters in our series of HH1 patients and healthy subjects.

## Discussion

Our study was aimed to evaluate the serum levels of iFGF23 and cFGF23 in HH1 patients without clinical evidence of organ dysfunction in respect to age-matched healthy subjects at baseline and one week after blood depletion due to phlebotomy and VBD respectively. The study, which was conducted while minimizing major confounding factors, including HH1-related complications [19] and chronic kidney disease (CKD) in which the reduced glomerular filtration leads to increased FGF23 levels [1], suggests that, at least in our cohort of HH1 patients, FGF23 dynamics appears largely independent of iron status and linked to erythrocyte parameters at baseline but not after phlebotomy. Moreover, while iron status markers (serum iron, serum ferritin and TSAT) showed the expected trend toward iron deficiency in healthy subjects after VBD [26, 32, 33], they remained near the upper normal value in HH1 patients after phlebotomy suggesting a persistent mild iron overload.

Phlebotomy/VBD may represent a condition of acute blood loss whose effects on *FGF23* transcription and subsequent cleavage have been reported to overlap those associated with iron deficiency [1, 8]. This was elegantly established by Rabadi and colleagues [27] who demonstrated that, in wild type mice, acute blood loss led to increased circulating levels of cFGF23 but not iFGF23. These changes occurred as early as 6 h following acute blood loss, were detected up to 48 h after the event, were associated with an increase of *Fgf23* mRNA transcription in the bone marrow (specifically in erythroid precursors) and were independent of iron deficiency and potentially dependent on EPO signaling. That EPO largely reproduces the effects of acute blood loss on FGF23 regulation was demonstrated in humans and mice after injection of rhEPO as also in different mouse models sharing increased endogenous serum levels of EPO as EPO-overexpressing and βT intermedia mice and mice treated with a HIF-proline hydroxylase (HIF-PH) inhibitor [6, 10, 27, 34, 35]. Keeping into account these data and the different iron status at baseline and after phlebotomy/VBD in our series of HH1 patients and healthy subjects, it is likely that the different dynamics of FGF23 observed in the two groups could be related to a different EPO response to blood depletion. Specifically, in healthy subjects, the coupled increased production and cleavage of FGF23, demonstrated by the increased serum levels of cFGF23 combined to the absence of changes of the serum levels of iFGF23, follows the increased production of EPO. The trend toward iron deficiency that may occur after VBD [36] may contribute to this feedback loop. In contrast, in HH1 patients, the trend toward persistent mild iron overload could act as a counter-regulatory mechanism of EPO production. This hypothesis is supported by data of the literature according to which iron opposes the physiologic effects of hypoxia [1, 37–39] likely through the enhancement of HIF-PH activity that targets HIF2α, a critical transcription factor stimulating the synthesis of EPO under hypoxic conditions [40, 41], for degradation [39]. In the absence of an EPO response, the dynamics of FGF23 does not modify and the serum levels of cFGF23, as also those of iFGF23, do not change after phlebotomy. However, serum levels of EPO were not measured in our study to prove or dispel this hypothesis and further studies are needed to resolve this issue.

Another important finding in our study is that the skeletal complications associated with HH1, which include osteoporosis, osteoarthritis, increased fracture risk and osteonecrosis [21–25], were not relevant findings in our cohort of HH1 patients. Indeed, both BMD and TBS as also the referred number of fractures and the VFA-MXA did not differ significantly in HH1 patients and healthy subjects. Up to one-third of HH1 patients develop osteoporosis and 40–80% develop osteopenia [22, 23, 25]. Jandl and colleagues provided the first insights into altered bone microarchitecture in HH1 using high-resolution peripheral quantitative computed tomography [24]. Iron overload is known to impact on bone health through the increase of bone resorption by facilitating osteoclastogenesis and increasing osteoclast activity, the reduction of osteogenic differentiation and the direct inhibition of extracellular matrix mineralization by direct inhibition of hydroxyapatite crystal growth [25, 42]. In *Hfe* knockout mice, bone loss, that is absent or mild under normal conditions, develops when animals are maintained on an iron-rich diet, implying that excessive iron loading is crucial to triggering bone loss [43]. It is likely that the comparable results observed in our cohort of HH1 patients and healthy donors reflect the criteria of selection of the HH1 patients, in particular the young age and the absence of complications, including cirrhosis, hypogonadism, diabetes and cardiomyopathy which are all important contributors of the impaired skeletal health in HH1 [23, 24].

The main strength of our study is that it is the first one in which the serum levels of FGF23 were investigated in HH1, including a parallel assessment of both iFGF23 and cFGF23 at baseline and after phlebotomy thus providing insights into FGF23 dynamics. The measured values, especially those obtained at baseline, could represent a reference for future studies. Moreover, by focusing on a cohort of patients without the typical complications associated with HH1, we minimized potential confounding factors and provided novel insights into the interplay between FGF23 and iron metabolism. However, we must acknowledge that being a pilot study it was adequately powered to detect large changes but had limited power for medium-sized effects. Therefore, some of non-significant changes should be interpreted cautiously. The absence of the measurement of the serum levels of EPO, especially after phlebotomy/VBD, and the short follow-up, that may not be sufficient to capture all possible homeostatic responses to phlebotomy, represent further limitations.

In conclusion, our pilot study is the first one to investigate the serum levels of iFGF23 and cFGF23 in patients with genetically proven uncomplicated HH1. The study includes a parallel assessment of both iFGF23 and cFGF23 at baseline and one week after phlebotomy with direct comparison with voluntary healthy blood donors.
